# The Effects of a Pilot Intervention for Community-Dwelling Adults with Rheumatoid Arthritis in Wuhan, China

**DOI:** 10.3389/fpubh.2013.00043

**Published:** 2013-10-10

**Authors:** Wenfang Deng, Jie Hu

**Affiliations:** ^1^Hope School of Nursing, Wuhan University, Wuhan, China; ^2^School of Nursing, The University of North Carolina at Greensboro, Greensboro, NC, USA

**Keywords:** rheumatoid arthritis, knowledge, self-efficacy, health-related quality of life, Chinese adults, China

## Abstract

This study examined the effects of a pilot educational intervention program on knowledge, perceived self-efficacy, and health-related quality of life (HRQoL) of community-dwelling adults with rheumatoid arthritis (RA). A convenience sample of 16 participants with RA completed the program in Wuhan, China. Data were collected in face-to-face interviews using questionnaires at baseline, post-test, and 1 month follow-up. Knowledge scores were significantly increased over time. Significant differences were found in pain self-efficacy, symptoms self-efficacy, bodily pain, social functioning, and role emotional functions. Community health providers should provide educational programs to improve HRQoL for adults with RA.

## Introduction

Rheumatoid arthritis (RA) reduces life expectancy about 3–10 years, especially when patients suffer the more severe forms of the disease (1). The World Health Organization (2) reports that the prevalence of RA ranges from 0.3 and 1%. It is more common in developed countries. The American College of Rheumatology (ACR) (3) reported that in developing countries, the prevalence of RA was significantly lower than in Northern Europe and North America (4, 5). However, Tobón et al. (1) have argued that the low prevalence of RA in developing countries may simply differ in reflect age distribution between developing countries and North America/Northern Europe. Further, Alamanos et al. (6) have noted that many patients with mild RA may not be diagnosed early in developing countries where access to medical care is limited, and this may lead to underestimation of the prevalence of RA in studies that rely on retrospective chart review.

In China, the rate of RA was 10.2% in 2008 compared to 8.6% in 2003. The prevalence of RA, however, varies in different regions. In 2008, rates of RA in urban and rural areas were 7.2 and 11.3%, respectively (7). The prevalence of RA in Nanning, Guangxi Province, is 0.27% in the Zhuang ethnic population and compared 0.28% in the Han ethnic population (8). Dai et al. (9) have reported that the prevalence of RA is 0.28% in Shanghai, China. In Shenzhen and Shandong provinces, the prevalence is higher than in other regions, at 0.44 and 0.40%, respectively (10, 11).

Millions of people with arthritis have limited knowledge about their disease and ways to manage the disease (12). Since no public awareness campaigns are delivering information on arthritis, patient education, and self-management programs are important (12). One study found that after completing the Program for Arthritis Control through Education and Exercise (PACE-Ex), participants showed significant improvements in self-efficacy to manage their arthritis, overall health status, and quality of life (13). Also, educational program conducted by Abourazzak et al. (14) found that RA patients’ function and quality of life remained stable for 3 years after the intervention. Similarly, Barlow et al. (15), found that the group provided with Arthritis Self-Management Program (ASMP) was significantly less depressed and had more positive moods. In addition, trends toward decreases in fatigue and anxiety were noted. The findings then suggested that the ASMP, when delivered in UK settings, was effective in improving perceptions of control, health behaviors, and health status (15). Clearly, patient education is a way to limit disability in rheumatoid diseases and to achieve improvements in quality of life (16).

In particular, patient education and intervention programs increased patient knowledge (17–19). Knowledge of the disease and its treatments is not certain to change behavior (20), but increasing knowledge is fundamental to the success of all educational interventions (21, 22).

Further, self-efficacy is thought to facilitate behavior change (12). A previous study indicated that self-efficacy perceptions play an important role in self-management activities, adoption and maintenance of health behavior changes, and positive health outcomes (23). Compared to personality traits that are generalized and relatively difficult to change, self-efficacy is potentially modifiable (24) and can be enhanced by an education intervention. In particular, psycho-educational programs can improve patients’ self-efficacy and thereby improve their ability to live with their RA (18).

Previous studies have shown that self-efficacy is associated with quality of life (25, 26). Cross et al. (26), for example, found that among patients with RA, those with higher self-efficacy reported better health status and lower overall costs. However, few studies in China have assessed the effects of educational intervention programs for patients with RA. It is important for Chinese adults with RA to be able to self-manage the disease in order to improve health. Therefore, the educational program examined here was aimed at improving health-related quality of life (HRQoL) for Chinese adults with RA. The study specifically examined the effects of this educational program on knowledge, perceived self-efficacy, and HRQoL of adults with RA.

The PRECEDE-PROCEED model (27) was used to guide this study. The PRECEDE-PROCEED model has nine phases. It provides a comprehensive structure of assessment and implement for health promotion intervention. Five phases are involved in the PRECEDE: social assessment, epidemiological assessment, behavioral and environmental assessment, educational and ecological assessment, administrative and policy assessment. In the PROCEED, there are four phases: implementation, process evaluation, impact evaluation, and outcome evaluation. These nine phases guide researchers in designing, implementing, and evaluating health promotion and other public health programs to meet the target population’s needs.

**Figure 1:**
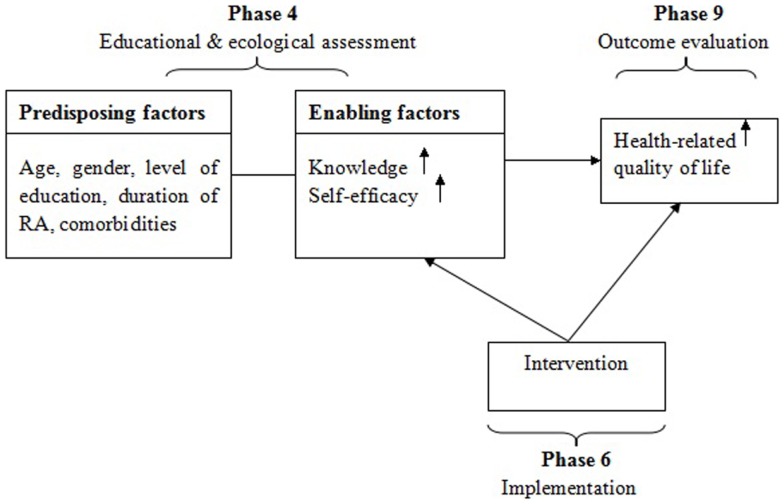
**PRECEDE-PROCEED Model (27) used in the educational program among Chinese adults with rheumatoid arthritis**.

In the present study, components of the model used in this study are educational and ecology assessment (Phase 4), implementation (Phase 6), and outcome evaluation (Phase 9). Before the intervention, predisposing factors (age, gender, level of education, duration of RA, comorbidities) and enabling factors (knowledge, self-efficacy, HRQoL) were assessed. Then the educational program of six weekly sessions was provided to the eligible participants. At the end of the last session and a month after the intervention, patients’ knowledge on RA, perceived self-efficacy, and HRQoL were evaluated. The increased knowledge and enhanced self-efficacy may have impact on HRQoL. The conceptual framework used to guide this study is presented in Figure 1.

## Materials and Methods

### Design

This pre-experimental study used one group with pre and post-tests to examine the effects of the educational program. Participants were recruited from one Community Health Center in Wuhan, China. Data were collected in face-to-face interviews at baseline, the end of the last session of the educational intervention, and a month after the intervention.

### Sample and setting

Participants with RA who resided in one district (Qingshan district) in Wuhan, China, were invited to participate in the educational program which was held in the Community Health Center (CHC). Located in central China, Wuhan is the capital city of Hubei province, with a population of more than 9.7 million (28). The population of Qingshan district is more than 0.48 million (28). The CHC in this district has five community health stations serving more than 42,000 residents. Intervention sessions and data collection were conducted in a meeting room of the CHC.

The criteria for inclusion were (1) participants met the 1987 ACR (formerly American Rheumatism Association) criteria for RA, the criteria were as follows: “(1) morning stiffness in and around joints lasting at least 1 h before maximal improvement; (2) soft tissue swelling (arthritis) of three or more joint areas observed by a physician; (3) swelling (arthritis) of the proximal interphalangeal, metacarpophalangeal, or wrist joints; (4) symmetric swelling (arthritis); (5) rheumatoid nodules; (6) the presence of rheumatoid factor; and (7) radiographic erosions and/or periarticular osteopenia in hand and/or wrist joints. Criteria 1 through 4 must have been present for at least 6 weeks. RA is defined by the presence of four or more criteria (referring participants’ health record and self-report), and no further qualifications or list of exclusions are required” [(3), p. 315); (2) aged older than 18 years; (3) understood, read and wrote Chinese; (4) did not attend any RA educational program in the past year; (5) clarified to time and place. The exclusion criteria were (1) having severe disability caused by RA; (2) severe comorbidities; (3) inability to complete questionnaires and participate sessions regularly; (4) cognitively impaired.

**Figure 2:**
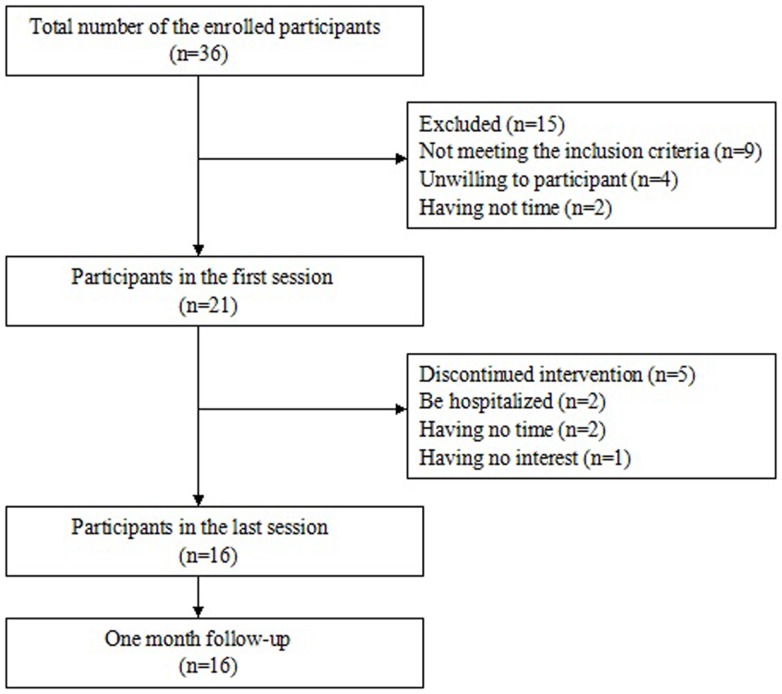
**Flow chart of the participants with rheumatoid arthritis in the study**.

A power analysis was performed to determine the sample size. In previous studies, effect sizes of self-efficacy in relation to pain and other symptoms are 0.45, 0.35, respectively (29, 30). The effect size was used 0.5 in this study. Based on the power analysis using an effect size of 0.5, a significance level of 0.05, and a power of 0.80 showed that a sample size of 30 was required (31, 32). The sample size was increased by approximately 15% to protect against the possibility of missing data. Therefore, 35 participants were needed in the study. A total of 36 interested individuals enrolled to participate. There were 21 eligible participants attending the first session. During the period of the intervention program, five persons discontinued to participate the intervention with two persons were hospitalized, two persons had time conflict, and one person had no interest in the program. At last, 16 participants completed the study after 1 month follow-up. Figure 2 presents the process of determining the sample.

### Intervention

The educational program included topics such as basic knowledge of RA (29, 33–35); effects and uses of medications (17, 36, 37); physical exercise (33, 34) pain management (33–35); joint protection and energy conservation (33–35); diet (34, 35). Detailed information on the intervention is given in Table 1.

**Table 1:** **Description of RA educational program**.

Session	Components of model	Purpose	Objectives	Content	Strategies
1	Increase knowledge Reinforce self-efficacy Improve HRQoL	Make sense of the basic knowledge of rheumatoid arthritis	Know the definition of rheumatoid arthritis Know the etiology, pathogenesis and pathology of rheumatoid arthritis	Introduce the purpose of the educational program and the researcher	Slides presentation, group discussion, reading materials, verbal encouragement
				Discuss several self-testing questions	
			Describe the clinical symptoms of rheumatoid arthritis Identify the classification of the severity of rheumatoid arthritis Know the diagnostic criteria of rheumatoid arthritis Know the related examination of rheumatoid arthritis Identify the differential diagnosis of rheumatoid arthritis	Definition of rheumatoid arthritis Etiology, pathogenesis and pathology of rheumatoid arthritis	
				Clinical symptoms of rheumatoid arthritis	
				Classification of the severity of rheumatoid arthritis	
				Diagnostic criteria of rheumatoid arthritis	
				Related examination of rheumatoid arthritis	
				Differential diagnosis of rheumatoid arthritis	
				Overview of this session	
				Consult and answer questions	
2	Increase knowledge Reinforce self-efficacy Improve HRQoL	Make sense of medications of rheumatoid arthritis	Know the standardized treatment of rheumatoid arthritis Identify the types of medication of rheumatoid arthritis Know the effects and side effects of medications Know the methods of taking medications	Overview of the last session	Slides presentation, Group discussion, Reading materials, Verbal encouragement
				Discuss several self-testing questions	
				Introduce the standardized treatment of rheumatoid arthritis	
				The types and effect of medication of rheumatoid arthritis	
				The effects and side effects of medications	
				The methods of taking medications	
				Overview of this session	
				Consult and answer questions	
3	Increase knowledge Reinforce self-efficacy Improve HRQoL	Promote healthily physical exercise	Know the importance of physical exercise Know the suitability of physical exercise Describe daily life training Describe the ways to do exercise	Overview of the last session	Slides presentation, group discussion, reading materials, group activity, demonstration, verbal encouragement
				Discuss several self-testing questions	
				Introduce the importance of physical exercise	
				The suitability of physical exercise	
				Introduce the methods of daily life training	
				Demonstrate how to do exercise	
				Overview of this session	
				Consult and answer questions	
4	Increase knowledge Reinforce self-efficacy Improve HRQoL	Master some methods to relieve pain	Know the method of keritherapy to relieve pain Know and master the method of sunbath to relieve pain Know the method of local heat therapy to relieve pain	Overview of the last session	Slides presentation, group discussion, reading materials, verbal encouragement
				Discuss several self-testing questions	
				The method of keritherapy to relieve pain	
				The method of sunbath to relieve pain	
				The method of local heat therapy to relieve pain	
				Overview of this session	
				Consult and answer questions	
5	Increase knowledge Reinforce self-efficacy Improve HRQoL	Master some methods to protect joint	Know the tips of keeping warm	Overview of the last session	Slides presentation, group discussion, reading materials, group activity, demonstration, verbal encouragement
			Describe some details to protect joint in daily life	Discuss several self-testing questions	
			Identify the importance of the balance of rest and exercise	Introduce the tips of keeping warm	
			Know some ways to regulate lifestyle	Some tips to protect joint in daily life	
			Describe some tips of appropriate housework	Use some ways to regulate lifestyle	
			Know the principles of joint protection	Some tips of appropriate housework	
			Describe some ways to protect joint	The principles of joint protection	
				Some ways to protect joint	
				Overview of this session	
				Consult and answer questions	
6	Increase knowledge Reinforce self-efficacy Improve HRQoL	Promote healthy diet for rheumatoid arthritis	Know the importance of healthy diet Describe some tips of healthy diet Identify the harmful food for rheumatoid arthritis Identify the beneficial food for rheumatoid arthritis Know six unfavorable aspects of diet for rheumatoid arthritis	Overview of the last session	Slides presentation, group discussion, reading materials, verbal encouragement
				Discuss several self-testing questions	
				Introduce the importance of healthy diet	
				Some tips of healthy diet	
				The harmful food for rheumatoid arthritis	
				The beneficial food for rheumatoid arthritis	
				Six unfavorable aspects of diet for rheumatoid arthritis	
				Overview of all the sessions and acknowledgment	
				Consult and answer questions	

Each intervention session lasted about 1 hour and was led by one of the authors. Educational strategies included powerpoint presentations, group discussions, group activities, reinforcement, and low-literacy and pictorial reading materials (38–40). Teaching strategies of simplification, repetition, repeating back, and multiple opportunities to ask questions were used to help participants understand and recall the recommended health behaviors in sessions. The content of the powerpoint consisted mainly of pictures combined with simple words. Group discussions were aimed at encouraging patients to share their opinions, what they had already changed, the benefits obtained and ask questions. Materials with the same content as the educational sessions were delivered to participants in each session.

### Procedure

The study obtained approvals from the university and the selected Community Health Center (CHC). Before the intervention, consent forms to the study were signed by participants. Before each class, the authors called participants to remind of the time and place so the participants would come to the classes on time. During the educational program, participants were given incentives such as towels, toothpastes, and toothbrushes to show appreciation for their participation in the study. Before each session, participants were offered educational materials associated with the sessions. To obtain family support, family members were encouraged to attend the program.

### Instruments

Instruments used in the study were the Demographic Questionnaire, the Patient Knowledge Questionnaire (PKQ), the Arthritis Self-Efficacy Scale (ASES), and the Medical Outcomes Study Short Form 36 (SF-36).

#### Demographic questionnaire

This questionnaire asks for data about age, gender, level of education, health insurance, duration of RA, comorbidities, marital status, persons living in household, family income, employment status, and smoking history.

#### Patient knowledge questionnaire in rheumatoid arthritis

The PKQ in RA (41) is designed to assure the accuracy of the patient’s information on RA and its treatments. The questionnaire consists of 16 questions in four subscales, each unit of items: (1) general knowledge of RA (maximum score 9); (2) drugs and how to use them (maximum score 7); (3) physical exercise (maximum score 7); and (4) joint protection and energy conservation (maximum score 7). The questions are multiple-choice. The maximum score is 30. Higher scores indicate more correct patient information. The Alpha coefficient on the scale was 0.72, indicating internal consistency, and test-retest reliability was 0.81 (41). The authors translated the English version of the PKQ into Chinese, and then two bilingual translators back translated the PKQ. Finally, one author and the author of the original questionnaire analyzed the content equivalence of the translation. Alpha coefficient of the Chinese version questionnaire was 0.61 in the current study.

#### Arthritis self-efficacy scale

The 20-item ASES was developed by Lorig et al. (42) to measure patients’ confidence in handling their arthritis pain, daily function, and other symptoms such as fatigue and frustration. Responses to the 20 items range from very uncertain (1) to very certain (10). The 20 items are divided into three subscales: (1) pain self-efficacy (PSE, five items); (2) function self-efficacy (FSE, nine items); and (3) other symptom self-efficacy (OSE, six items). The present study did not include the nine questions on function (FSE), because the function self-efficacy scale is not applicable to the Chinese. Self-efficacy was indicated by two scores: one for pain (PSE) and one for other symptoms (OSE). Cronbach’s alphas for the two subscales were 0.75(PSE) and 0.87(OSE) (42). Test-retest reliabilities of the two subscales were 0.87(PSE) and 0.90(OSE) (42). The authors translated the English version of the ASES into Chinese. Then two bilingual translators back translated the tool. Finally, one author and a native speaker analyzed the content equivalence of the translation and the back translation. Cronbach’s alphas for the two subscales in the Chinese version were 0.88(PSE) and 0.91(OSE) in the current study.

#### Medical outcomes study short form 36

The Medical Outcomes Study Short Form 36 (SF-36) is a 36-item instrument designed to measure HRQoL (43). The 36 items are divided into eight subscales: physical functioning (PF), physical and emotional roles (RP and RE), bodily pain (BP), general health (GH), vitality (VT), social functioning (SF), and mental health (MH). Physical health domains include PF, RP, and BP, while MH domains include SF, RE, and MH. VT and GH contain both physical and mental components. Scores in each subscale range from 0 to 100, with higher scores indicating better health status. Each dimension of the SF-36 has an alpha greater than 0.80, except for SF (*a* = 0.76) (44). The SF-36 scales have been found reliable (intra-class correlation coefficients 0.76–0.93) among patients with RA (45). Cronbach’s coefficient alpha for the Chinese version of the SF-36 in Chinese-speaking patients with RA was 0.92, indicating excellent internal consistency (46). In the current study, Cronbach’s alpha for the Chinese version of the SF-36 was 0.78.

### Data analysis

Descriptive statistics were used to analyze the demographic data. The categorical variables were described using frequency and percentage and the continuous variables were described using mean and standard deviation. The repeated measures ANOVA with Tukey HSD for pairwise comparison was conducted to compare pretest and post-test and 1 month follow-up variables. A significant level of 0.05 was used to test for significance.

## Results

### Characteristics of the participants

A total of 21 participants who met the study inclusion criteria were recruited at baseline and 16 completed the study. The mean age of participants was 64.81 years (SD = 7.88), with a range from 47 to 76 years. Most participants were female (87.5%) and had a primary school education or more (87.5%). The majority (87.5%) were married, retired (87.5%), and living with a spouse (62.5%). More than half of the participants had a family monthly income of RMB 1000–2000 or less; half of the participants had health insurance. The average length of time since a diagnosis of RA was 9.37 years (SD = 5.02). More than half of the participants (56.2%) had comorbidities (e.g., hypertension, diabetes, heart disease, gout, and osteoarthritis). Only 18.8% had a smoking history. The detailed information of the participants is presented in Table 2.

**Table 2:** **Demographic characteristics of the participants (*N* = 16)**.

Variables	*N*	%
Gender
Male	2	12.5
Female	14	87.5
Comorbidities
Heart disease	2	12.5
Diabetes	1	6.3
Hypertension	5	31.3
Gout	3	18.8
Osteoarthritis	3	18.8
None	7	43.8
Health insurance	8	50.0
Education level
No formally educated	2	12.5
Primary school	5	31.3
Junior middle school	5	31.3
Senior middle school	1	6.3
College or above	3	18.8
Marital status
Married	14	87.5
Widowed	2	12.5
Employment status
Working full or part-time	2	12.5
Retired	14	87.5
Income status
<1000	6	37.5
1000–2000	8	50.0
2000–3000	2	12.5
>3000	0	0
Persons living in household
Spouse and Children	5	31.3
Spouse	10	62.5
Children	1	6.3
Smoking history	3	18.8

### Description of RA knowledge, self-efficacy and quality of life

Descriptive statistics were used to describe RA knowledge, arthritis self-efficacy for pain, arthritis self-efficacy for other symptoms, and scores on the SF-36 (PF, RP, BP, GH, VT, SF, RE, and MH) at three points. The means and standard deviations (SD) of the variables are presented in Table 3.

**Table 3:** **Repeated measures ANOVA examining the effects of the intervention on rheumatoid arthritis knowledge, arthritis self-efficacy, and health-related quality of life over three times (*N* = 16)**.

Variables	Mean Square	df	*F*	*P*	Baseline Mean (SD)	Post-intervention Mean (SD)	1 month follow-up Mean (SD)
Knowledge	338.40	2	61.95	0.000*	8.38 (3.52)	17.50 (4.37)^a^	13.94 (3.42)^b^
Arthritis self-efficacy: pain	1.76	2	2.53	0.096	6.05 (0.80)	6.71 (1.15)^a^	6.41 (1.02)
Arthritis self-efficacy: other symptoms	1.28	2	3.45	0.045*	6.24 (0.66)	6.56 (0.82)	6.80 (0.92)^b^
Physical functioning	13.91	2	0.99	0.383	43.09 (7.83)	42.43 (5.92)	41.25 (4.98)
Role physical	11.63	2	0.37	0.694	43.23 (7.43)	42.62 (9.17)	44.30 (8.53)
Bodily pain	216.98	2	7.88	0.002*	37.90 (5.35)	43.26 (6.83)^a^	44.95 (5.77)^b^
General health	64.59	2	2.74	0.081	35.00 (6.27)	38.93 (9.30)	36.25 (8.82)
Vitality	7.51	2	0.30	0.743	52.29 (7.85)	53.07 (10.11)	51.70 (8.29)
Social functioning	91.13	2	2.20	0.129	43.90 (6.57)	46.28 (9.01)	48.67 (7.45)^b^
Role emotional	136.30	2	1.98	0.156	35.47 (8.46)	41.30 (8.92)^a^	38.14 (12.04)
Mental health	20.52	2	0.55	0.581	48.25 (8.22)	48.60 (10.49)	50.36 (8.35)
Physical component summary	6.00	2	0.52	0.599	40.40 (5.09)	41.39 (5.00)	41.51 (3.64)
Mental component summary	49.67	2	1.54	0.231	45.58 (7.86)	48.75 (10.60)	48.49 (7.84)

*Represented that the scores were significant at the level of 0.05.

^a^*p* < 0.05, baseline versus post-intervention using Tukey HSD for pairwise comparisons.

^b^*p* < 0.05, baseline versus 1 month follow-up.

Knowledge was measured by the PKQ in RA (PKQ). As shown in Table 3, the knowledge score of the participants was 8.38 (SD = 3.52) at baseline, and this score increased to 17.50 (SD = 4.37) after the intervention, but dropped to 13.94 (SD = 3.42) at 1 month follow-up. Mean scores on arthritis pain self-efficacy were 6.05 (SD = 0.80) at baseline, 6.71 (SD = 1.15) after the intervention, and 6.41 (SD = 1.02) at 1 month follow-up (Table 3). The mean scores on self-efficacy for symptoms were 6.24 (SD = 0.66) at baseline, 6.56 (SD = 0.82) after the intervention, and 6.80 (SD = 0.92) at 1 month follow-up (Table 3). HRQoL was measured by the SF-36. Table 3 shows the scores of the components of HRQoL at three times.

### Intervention effectiveness

Repeated measures ANOVA with Tukey HSD for pairwise comparisons was used to examine differences in RA knowledge, arthritis self-efficacy, and scores on the SF-36 at the three measurement points.

At baseline, participants were unable to correctly answer 50% or more of the questions on the knowledge test. There were significant improvements in scores over time (*F* = 61.95, df = 2, *p* = 0.000) (Table 3). Tukey HSD Pairwise comparisons of knowledge showed significant improvement in the knowledge score from pre-to post-test, pre-to 1 month follow-up and post-test to 1 month follow-up. It indicates that the educational program significantly improved arthritis knowledge although the sample size is small.

The tests of within-subjects effects of pain self-efficacy revealed no significant differences over three times (*F* = 2.53, df = 2, *p* = 0.096) (Table 3). However, pairwise Tukey HSD comparisons of pain self-efficacy over the three times showed a statistically significant difference between baseline and post-test (*p* = 0.01), though no significant difference was shown at 1 month follow-up (*p* = 0.18). This may indicate that the program enhanced the participants’ confidence in coping with pain to some extent even though aggravated pain in the later period may decrease the self-efficacy. For other symptoms self-efficacy, tests of within-subjects effects showed significant difference over three times (*F* = 3.45, df = 2, *p* = 0.045) (Table 3). Tukey HSD pairwise comparisons of other symptoms self-efficacy also revealed a significant difference from baseline to 1 month follow-up (*p* = 0.036). Thus, after attending the educational program, participants have more confidence to manage their arthritis effectively.

The SF-36 scores for PF, RP, GH, VT, and MH remained relatively stable over time, and there was no significant difference in these five domains (Table 3). Only tests of within-subjects effects of BP showed significant improvements over the three measurement times (*F* = 7.88, df = 2, *p* = 0.002). However, Tukey HSD pairwise comparisons showed significant differences in BP, SF, and role emotional over the three measurement times. A significant improvement in BP was revealed at post-test (*p* = 0.009) and 1 month follow-up (*p* = 0.001), indicating that participants felt less pain than before, after attending the educational program. A significant difference was also found in SF at 1 month follow-up (*p* = 0.043), though no difference was observed at post-test (*p* = 0.323). There was no significant difference in role emotional functioning at 1 month follow-up (*p* = 0.458), though a significant improvement was observed at post-test (*p* = 0.016). The mean scores of the physical component summary and mental component summary were 40.40 (SD = 5.09) and 45.58 (SD = 7.86) at baseline. Both scores increased at post-test and 1 month follow-up, however, there were no significant difference in physical health and MH over the three times. Only few domains of the SF-36 scores presented significant improvement.

## Discussion

This study examined the effects of a group educational program on knowledge, self-efficacy and HRQoL among community-dwelling adults in China with RA. It was expected that participation in the educational program would lead to improved knowledge, self-efficacy and HRQoL. Despite the small sample, knowledge scores significantly increased at the post-test, and maintained at 1 month follow-up. Self-efficacy for pain improved after the six weekly intervention sessions. The scores of other symptoms self-efficacy did not significantly increase at post-test, but a significant difference was found in self-efficacy for other symptoms at 1 month follow-up. As for the SF-36 quality of life scores, BP scores improved both at the post-test and 1 month follow-up; SF improved after 1 month follow-up; and role emotional functioning improved at post-test.

### Effects of the educational intervention on RA knowledge

The improvements of participants’ knowledge at post-test and at 1 month follow-up are consistent with a systematic review conducted by Niedermann et al. (18), in which educational programs improved knowledge, and the influence was long-term. Some previous studies have also reported similar findings (14, 47–49).

This study emphasized the need to increase the knowledge of RA among participants. All the participants volunteered to attend the educational program, which indicated that they had need for more information about their disease. Educational programs about RA are seldom provided at CHCs; in addition, doctors and nurses in hospitals do not have time to give detailed information about the disease to patients. Therefore, this program was well attended by participants. During the sessions, the participants listened carefully and discussed their problems with the authors and others. Before every session, the first author reviewed the content taught in the last session and she gave a summary at the end of a session. Effective teaching strategies, including simplification, repetition, repeating back, and multiple opportunities to ask questions to help participants understand and recall the recommended health behaviors, were used in the sessions. In addition, materials related to the content of the sessions also helped to strengthen knowledge. These strategies may explain the increase of knowledge among participants. In this study, the scale assessing the score of knowledge has items on medical knowledge about RA, and most of the participants are older adults and the score may be decreased over time. These may explain the reasons why the knowledge score dropped 3.56 from post-intervention to 1 month follow-up.

### Effects of the educational intervention on self-efficacy

Following the educational program, participants demonstrated significantly greater arthritis self-efficacy for pain. In addition, the level of arthritis self-efficacy for other symptoms increased at 1 month follow-up. The positive effects on arthritis self-efficacy found in this study, are in accordance with those reported in previous evaluations of patient education programs for people with RA (18, 49, 50).

Participants had more confidence to handle their pain and other symptoms of RA effectively. By attending the program, they not only obtained more information about the disease, but also peer support from each other. They realized that they were not the only person who had this disease and that many persons were in the same condition as themselves. They discussed common experiences and exchanged coping strategies with one another and consulted about their questions with the first author, which may have enhanced their confidence in managing the disease. The emphasis on coping strategies and appropriate self-care behaviors in sessions, together with the group interactions, probably improved self-efficacy.

### Effects of the educational intervention on health-related quality of life

Rheumatoid arthritis is progressive in nature. Therefore maintenance of HRQoL over time can be regarded as a positive outcome (29). In this study, BP improved significantly both at the post-test and 1 month follow-up; SF improved after 1 month follow-up; and role emotional functioning improved at post-test. No significant difference was found in the other domains of the SF-36 quality of life.

Patient education has been considered as one way to limit disability in rheumatic diseases and improve quality of life (16). Effects on HRQoL, however, may not show up for a short period of time. For example, change in depressive symptoms may take longer than 10 weeks to manifest (29). It is difficult to find significant improvements in all domains of the HRQoL in the short term. Future studies are needed to evaluate the effects of RA educational programs held in communities in a long-term.

### Limitations and implications for future research

This study had several limitations. First, the sampling method was convenience sampling and the sample size was very small, particularly several participants dropped out the study, which may have limited the representativeness of the sample and the generalizability of the findings. Study findings should be explained with caution. Also, because the participants in the program were volunteers, they may have attached more importance to self-care and more actively responded to the disease than patients who did not participate in this study. Second, the present pilot study had a lack of a control group to confirm the effects of the educational program. Third, the follow-up time was relatively short, the study did not examine the long-term effects of the educational program on participants’ knowledge, self-efficacy, and HRQoL.

In future studies, a randomized control group is needed to confirm the positive benefits of attending the educational program. In addition, long-term follow-up evaluations should be conducted to determine whether changes are maintained over time. Finally, future studies should expand the sample size to make the results be generalizable.

### Implications for practice

The positive results found in the present study provide evidence of the importance of group educational programs for improving knowledge of RA, self-efficacy, and some aspects of HRQoL among community-dwelling adults with RA. The benefits attained by attending the educational program indicate that such programs are worthy of further exploration. Community health providers should provide educational programs to adults with RA. In addition, the strategy of group discussion provides patients opportunities to share experiences and exchange coping strategies in educational programs. The teaching strategies of simplification, repetition, repeating back, and multiple opportunities to ask questions to help patients understand and recall the recommended health behaviors are also very important.

## Conclusion

In conclusion, this educational program had positive impacts on knowledge, self-efficacy, and some aspects of HRQoL among Chinese community-dwelling adults with RA. Through the program, persons with RA mastered some methods of physically exercising, protecting joints, relieving pain, and consuming a healthy diet, which improved their HRQoL. However, randomized controlled studies with longer follow-up evaluations are needed to confirm the benefits derived from the educational program.

## Conflict of Interest Statement

The authors declare that the research was conducted in the absence of any commercial or financial relationships that could be construed as a potential conflict of interest.
